# Dropsy, and the Pathological Conditions That Give Rise to It

**Published:** 1863-02

**Authors:** N. S. Davis

**Affiliations:** Attending-Physician


### MEDICAL WARDS OF THE MERCY HOSPITAL.

### DROPSY, AND THE PATHOLOGICAL CONDITIONS THAT GIVE RISE TO IT.

By N. S. DAVIS, M.D., Attending-Physician.

Read before the Chicago Medical Society.

During the recent course of clinical instruction in the Mercy Hospital, a series of cases were presented to the class, which strikingly illustrate both the various pathological conditions giving rise to dropsical effusions, and the differences in the location and progress of such effusions. Excluding hydatids, or cystic dropsy, we may arrange all other cases into three classes, viz.:—1st. Those that arise directly from inflammation;

2d. Those dependent on mechanical obstructions in some part of the vascular system; 3d. Those arising from alterations in the blood.

Of the first class I shall not speak at present, although we have on our list two very interesting cases,—the one of pleurisy, and the other of pericarditis, both accompanied by copious serious effusion.

The second class of cases, includes all such as depend on mere mechanical obstruction, whether from the pressure of tumors, the gravid uterus, ligatures, fibrinous coagula, diseased valves, or enlarged viscera. But those of chief interest, arise from either valvular disease of the heart, or from diseases of the liver and spleen.

Case I.—Mrs. J., an American woman, aged about 50 years; was admitted into the medical wards of the Mercy Hospital in October, 1862.

She was considerably emaciated; countenance expressive of anxiety, with a slight œdema of the eye-lids; pulse 85 per minute, small, and moderately firm; tongue clean; bowels regular; appetite fair, but digestion imperfect; secretion of urine very scanty, becoming turbid after standing, from an excess of phosphatic salts, but yielded no evidence of containing albumen or sugar. Her respiration was short, and accompanied by a sense of oppression, which was so much increased by the recumbent position, that she maintained the sitting posture all the time, both night and day. The cellular tissues of the lower part of the body, and the whole lower extremities, were greatly distended with œdematous infiltration. A small amount of effusion had also accumulated in the cavity of the peritonium. The œdema of the legs had existed several months; and the over-distended skin on the calves of the legs had become broken, and the serum was escaping in such quantity as to keep the feet and ankles constantly wet.

On making a careful physical examination, no evidence of disease was found in any of the abdominal viscera; but over the cardiac region, there was increased extent of dulness, and a loud, rough, bellows murmur, covering all the space between the first and second sounds of the heart, showing plainly diseased valves with hypertrophy of the heart. On tracing the history of the patient, it was evident that the cardiac disease, in this case, originated at least ten years previous, from an attack of sub-acute rheumatism. It will be observed that the dropsical effusions, in this case, were most marked in the parts most distant from the heart, and most dependent; and this is true of all cases dependent on mechanical obstruction in the central organ of the circulation.

CASE II.—Mrs. C., born in Ireland, the mother of several children, was admitted into the Mercy Hospital in December, 1862; aged about 38 years.

At the time of admission, she was emaciated; countenance expressive of sadness or despondency; face pale; lips and, indeed, skin generally bloodless, presenting a strongly anemic look; skin cool; pulse soft, and about 90 per minute; respiration shorter and quicker than natural; poor appetite, and food often becoming sour, and sometimes being rejected; bowels irregular, being sometimes costive, and sometimes too free; urine very scanty, and depositing a large amount of phosphatic and lithic acid salts, but containing no albumen. On examining the abdomen, it was found greatly distended by an accumulation of serous fluid in the cavity of the peritoneum, accompanied by a considerable enlargement of the liver and spleen. There was no tenderness or other indications of inflammatory action. For the last few days, the ankles have been also slightly œdematous; aside from which, the dropsical appearances are confined exclusively to the abdomen. There were no signs of disease in the viscera of the thorax or pelvis.

The history of this case showed, that the patient had been attacked with intermittent fever during the past summer, which was somewhat protracted and accompanied by pain in the hypochondriac regions, and much disorder of the digestive organs. Although the intermittent paroxysms ceased, and have not returned during the last three months, yet she did not acquire good health. Her bowels were more or less costive; digestion imperfect and flatulent; muscular weakness; countetenance sallow; and urine scanty and turbid.

About six weeks since, the abdomen began to enlarge, and has steadily increased until attaining its present great distension. In this case, it is evident that the dropsical effusion is chiefly the result of direct mechanical obstruction to the portal circulation, by the enlargement of the liver; and, hence, instead of the dropsical effusion being controlled by distance from the heart and gravitation, as in Case I., it is limited to the cavity containing the distribution of the obstructed bloodvessel; and no mere change of position of the patient produces any change in the location of the effusion.

Case III.—Mr. W., an American, aged 43 years, was admitted into the Mercy Hospital, Nov. 30th, 1862. He was somewhat addicted to the usc of intoxicating drinks, and eight or nine months previous to admission, had been exposed to a thorough wetting and cold, which was followed by some pain in the right side, and slight fever. These symptoms lasted only a few days, but they were followed by indigestion, flatulency, constipation, and general feelings of ill-health, but not sufficient to confine him to his house. About six weeks since, he noticed the commencement of some enlargement of his abdomen, which continued steadily to increase until his admission to the hospital. At that time he was considerably emaciated; his lips pale and thin; his pulse small, tense, and about 100 per minute; skin dry and natural in temperature; appetite variable; bowels inactive; urine very scanty, but destitute of albumen; and his abdomen so much distended with fluid as to impede the descent of the diaphragm, rendering respiration short, and producing much uneasiness from mechanical pressure. No œdema of the extremities, or of any part of the cellular tissue. No solid tumor could be felt in any part of the distended abdomen, but the accumulation of fluid in the peritoneal cavity was so great as to render it difficult to define the lower boundaries of the liver and spleen.

By careful percussion, however, a line of tympanitic or intestinal resonance was found to extend transversely across the right hypochondriac and epigastric regions, quite under the margin of the ribs. This showed that the transverse colon was crowded upward by the fluid in the peritoneal cavity, and occupied the place usually rendered dull by the lower margin of the liver, when of its natural size. This rendered it very probable that the liver, instead of being enlarged, was contracted, or in a state of cirrhosis. Following the line of the colon to the left side, it became more obscure and deflected downwards, as if crowded in that direction by some pressure from above. This fact, together with uniform dulness and general fulness of the left hypochondriac region, left but little doubt that the spleen was moderately enlarged. The results of a careful physical examination, compared with the history of the case, led us confidently to diagnosticate the case as one of well developed *cirrhosis* of the liver, coincidently, with moderate enlargement of the spleen.

The contracted condition of the liver, greatly interfering with the circulation through the hepatic branches of the vena porte, was, doubtless, the immediate cause of the dropsical effusion. Hence, as in Case II., it takes the form of circumscribed dropsy, limited to the abdominal cavity, and is not influenced by change of position, on the part of the patient, or by gravitation. Finding remedial agents to exert very little influence over the progress of this case, and the patient suffering much from mechanical distension of the abdomen, he was tapped by Prof. HOLLISTER, the attending-surgeon of the hospital, and about three and a-half gallons of limpid serum drawn off. In about three weeks, the effused fluid had re-accumulated to such an extent that tapping was again resorted to, and between three and four gallons of fluid again discharged. At about the same intervals, a third and fourth tapping became necessary. Of course, so rapid and copious a drain of serous fluid from the blood, produced rapid exhaustion of the patient. About one week after the last tapping, symptoms of a low grade of peritoneal inflammation supervened, and the patient died in a few days.

*Post mortem.*—Twenty-four hours after death, a *post mortem* examination was made. On opening the abdomen, about two gallons of turbid serum escaped. The whole surface of the peritoneum, both that lining the abdominal walls and covering the intestines, was minutely injected, of a dark red color, and covered in patches with a white and partially organized membranous exudation, which possessed so little tenacity, however, that it is hardly proper to say it constituted a bond of adhesion. The spleen was found nearly double its natural size and weight, color nearly natural, and texture firm. The liver was found firmly adherent, at several points, to the ribs and parts surrounding it, as the result of former inflammation. Its color was a shade lighter than natural, and its size diminished nearly one-half. Its entire surface presented a knotted or lobulated appearance, well represented by the section of the central portion of the right lobe, which is here presented for examination. The color has faded by masceration; but the contraction of the lobules, and the "hob-nail" appearance is strongly exhibited. The texture of the organ was firm; and the gall-bladder moderately filled with bile. All other important organs were in a healthy condition. Here we have a well-marked case of cirrhosis, or contraction of the liver leading to the same results, so far as the dropsical effusion is concerned, as in Case II., in which, the same organ had undergone great enlargement. The two cases, though directly opposite in several respects, yet presented in common, the one pathological condition which was the immediate cause of the dropsy, namely, mechanical obstruction in the hepatic branches of the portal vein.

The third-class of cases of dropsy, which arise from alterations in the proportion of the constituents of the blood, we shall illustrate by only one case in detail:—

Case IV.—Mr. M., native of Ireland, was admitted into the Mercy Hospital in January, 1863. He was, naturally, a large, muscular man, accustomed to physical labor. He stated that his health and strength had been failing several months, accompanied by indigestion, constipation, mental despondency, dull pains in the loins, and weakness of the lower extremities. He had been somewhat addicted to the use of alcoholic drinks. About three weeks before he came to the hospital, he began to exhibit a bloated aspect generally; but the swelling was most marked in the feet and legs, after being up through the day. This swelling, or general œdema, rapidly increased, until he was obliged to take to his bed. On admission to the hospital, his skin was dark, approaching a bronzed hue; surface cool; pulse small, and 95 per minute; mental faculties, apparently, dull; bowels costive; and the whole exterior surface much bloated from œdematous infiltration; but the swelling was much more marked in the parts most dependent.

This was illustrated, not only by the position of the extremities, but the left side of the trunk of the body, on which he had been lying several hours: would pit, on the application of pressure, to the depth of half an inch, while on the opposite side, the pitting would be comparatively slight. There was also a small amount of effusion into the cavity of the abdomen, but none in the chest. The amount of urine secreted was much less than natural, but containing so much albumen, that, on the application of heat or nitric acid, the white flaculent precipitate rendered the whole mass thick in the test-tube. The past history of this case,—the present symptoms, and the condition of the urine,—render it quite certain that the patient has granular disease, or degeneration of the kidneys, frequently called: "Bright's disease." A fair specimen of that form of disease is presented by this kidney, which I have brought for the examination of the Society. In such cases as this, the large amount of albumen constantly being excreted through the kidneys, soon reduces the relative proportion of albumen in the whole mass of the blood, thereby, rendering it much less viscid than natural. When this process of lessening the viscidity of the blood is carried beyond a given point, the relation between it and the capillary vessels is so changed that the physical law of exosseous predominates, and effusion or infiltration of the watery element of the blood necessarily takes place. If, at the same time that the albumen is escaping through the kidneys, the amount of water eliminated through both the kidneys and cutaneous surface is greatly diminished below the natural standard, the relative proportion of the constituents of the blood is so rapidly altered that dropsical symptoms may be developed in twentyfour or thirty-six hours; as we see following sudden congestions of the secreting structure of the kidneys from exposure to cold and wet, and during the convalescence from eruptive fevers. But the viscidity or density of the blood may be as much diminished by a loss of red corpuscles as by the escape of albumen. Hence, excessive hemorrhages, suppressed menstruation, diseases of the spleen, and the influence of malaria, are all capable of, so far, diminishing the proportion of red corpuscles, as to develop general dropsical effusions.

Thus far, we have directed attention to the essential pathological conditions producing dropsy, either by obstructing the natural flow of the blood, or by changing the relative proportion of its constituents; but in a therapeutic aspect, it is almost equally important, that we appreciate correctly the condition of the vital properties of the solid textures. Diminution of vital affinity,—that property by which the organic atoms of the various tissues are held in proper proximity and relationship with each other,—necessarily diminishes the tonicity and contractility of all the muscular, vascular, and secretory structures; and, hence, not only retards secretion and capillary action, but directly favors permeation of tissues or dropsical effusions. The presence of this pathological condition of the solids in many cases of dropsy, led the older writers to divide all dropsical effections into two classes, viz.: sthenic and asthenic, or active and passive; and though subsequent pathological investigations have rendered the ancient classification obsolete, they have by no means diminished the necessity of appreciating the general condition of the vital properties as carefully as the more local lesions, if we would direct the treatment with the highest degree of success. This was well illustrated in the treatment of the second case, already related. The dropsical effusions being regarded as dependent on the enlargements of the liver and spleen, coupled with considerable impoverishment of the blood; she was treated, for three weeks, by mercurial alteratives, iodine preparations, diuretics, and quinine, with very little benefit; indeed, these remedies entirely failed, either to increase the secretion of the skin and kidneys, or to materially reduce the visceral enlargements. Suspecting the difficulty to arise from loss of tonicity, or, more properly, vital affinity and contractility in the capillary and secretory structures, we directed her to be put upon the use of strychnine and citrate of iron, in doses of one-sixteenth of a-grain of the first with three grains of the last, four times a-day. Very little change was observable during the first three or four days; but by the middle of the second week, after commencing this treatment, it was found that the patient was urinating freely, the dropsical appearances were much diminished, and her strength improved. The same treatment was continued; and in four weeks the patient had entirely recovered,—there remaining neither dropsical effusions nor visceral enlargements.

The important practical inferences to be drawn from the foregoing cases and observations are: 1st. That dropsy is, in a pathological sense, not a disease but a symptom directly dependent on either inflammation, mechanical obstruction of blood-vessels, or altered composition of the blood. 2d. That the two latter conditions may arise from a great variety of primary pathological changes, both local and general. 3d. That all rational treatment of dropsical symptoms must depend on a clear appreciation of the primary pathological changes from which they have originated, compared with the general condition of the vital properties or forces.

We might illustrate these positions by a great variety of clinical cases and facts, but the details would doubtless, be tedious to the Society.

